# The Dengue Vaccine Dilemma: Balancing the Individual and Population Risks and Benefits

**DOI:** 10.1371/journal.pmed.1002182

**Published:** 2016-11-29

**Authors:** Jacqueline Deen

**Affiliations:** Institute of Child Health and Human Development, National Institutes of Health, University of the Philippines, Manila, Philippines

## Abstract

In a Perspective, Jacqueline Deeen discusses challenges in balancing the individual and population risks and benefits for CYD-TDV (Dengvaxia), the first available dengue vaccine.

In tropical Asia and Latin America, dengue is a significant public health threat. Several characteristics of the disease contribute to this high sense of priority, including its geographic spread, lack of specific treatment other than supportive therapy, and the burden it imposes on clinics and hospitals. In hyperendemic areas with circulation of the four dengue serotypes, recurrent dengue infections are common. Primary infection is generally asymptomatic or manifests as self-limiting dengue fever, whereas there is an increased risk of severe dengue during secondary infection [1]. The most prominent feature of severe dengue (previously called dengue hemorrhagic fever and dengue shock syndrome) is a transient increase in vascular permeability, resulting in plasma leakage that may lead to circulatory compromise, shock, and death [2]. Coagulation abnormalities, hepatitis, renal failure, myocarditis, or encephalitis may also occur. Postsecondary infections are associated with a reduced risk of disease [3].

Wide-scale dengue vaccination would represent a major advance in the control of the disease, but dengue vaccine development has been slow and difficult [4]. Vaccine development is hindered by the complex immune response to the dengue virus and the difficulty in eliciting concomitant protection against its antigenically distinct serotypes. CYD-TDV (Dengvaxia, Sanofi Pasteur) is the first dengue vaccine to become available, but it arrives with a complex set of challenges [5].

The CYD-TDV vaccine consists of four chimeric viruses made by replacing the premembrane and envelope structural genes of the attenuated yellow fever 17D vaccine strain with the corresponding genes from each of the four dengue serotypes [4]. CYD-TDV is administered subcutaneously as a primary dose followed by subsequent doses 6 and 12 months later. Large Phase III trials were conducted among 2 to 14 year olds in five Asian countries [6] and 9 to 16 year olds in five Latin American countries [7]. In pooled analysis of follow-up to 25 months after the first dose, vaccine efficacy against symptomatic dengue was 60% for all participants, 66% for those 9 years of age or older, and 45% for those younger than 9 years of age [8]. Importantly, among the Asian children vaccinated at ages 2 to 5 years, a statistically significant increased risk of hospitalized dengue was seen in the vaccine recipients. Subgroup analysis showed higher rates of protection among participants who were already seropositive prior to vaccination (i.e., partially dengue immune) compared to those who were not. CYD-TDV has been licensed in Paraguay, Mexico, Brazil, El Salvador, Costa Rica, and the Philippines for use in preadolescents, adolescents and adults from 9 to 60 years of age living in dengue-endemic areas [9].

The World Health Organization convened a Strategic Advisory Group of Experts (SAGE) who reviewed the evidence and recommended that countries consider introduction of the vaccine only in settings with high endemicity, defined by a seroprevalence of at least 70% in the target age group [10]. The experts advised not to use the vaccine in populations with seroprevalence <50%. The seroprevalence threshold was based on the modeling study published in this week’s *PLOS Medicine*, which used the assumption that CYD-TDV vaccination immunologically primes seronegative recipients, causing their first natural dengue infection to have higher severity, like that seen with secondary infection in unvaccinated persons [11]. The mathematical model predicts the greatest impact in a high endemicity setting where routine vaccination of 9 year olds at 80% coverage would reduce dengue-related hospitalizations by 13% to 25% over 30 years. In contrast, vaccination in low-transmission settings with a high population of seronegatives will increase the number of hospitalized dengue cases [11,12].

The WHO recommendations have split the dengue community into those that support [13,14] and challenge [15,16] them. In theory, wide deployment of CYD-TDV within the recommended populations could result in considerable public health benefit. But what of the seronegative individuals who are vaccinated and become at increased risk for severe disease? It has been argued that in high-transmission settings, CYD-TDV vaccination simply accelerates the natural progression of dengue disease acquisition by the population. That is, even if CYD-TDV primes seronegative individuals for a secondary-like infection associated with more severe disease, unvaccinated seronegative individuals would similarly experience secondary infections because of a high level of exposure [14]. If policymakers accept this argument and the decision to rollout CYD-TDV is made, how can this be done? Identifying the population that would benefit most is important not only to limit the negative impact of vaccination of seronegatives but also to ensure cost-effectiveness. Vaccination of populations who have already experienced secondary and tertiary dengue infections would be a waste of resources. Rational decision making requires reliable seroprevalence data, which are not easy to come by. Seroprevalence surveys are time-consuming, expensive, require technical expertise and their results are dynamic. The plaque reduction neutralization test (PRNT) is the most widely accepted approach for detecting and measuring dengue antibodies, but it is cumbersome, not widely available, and compromised by several limitations, including a wide variation in titer results in response to different testing conditions [17,18]. Simpler serological tests would be more practical but require further validation for this purpose. Other markers of dengue prevalence that rely on routine surveillance, such as mean dengue hospitalization age, are more accessible but of little use, as the relationship between this indicator and the optimal age of vaccination is not known. Individual screening using a simple, indirect dengue immunoglobulin G (IgG) test prior to immunization has been recommended [15]. Although this strategy may be feasible in some countries or in the private sector, the SAGE experts did not consider this approach generally advisable because of the limitations of available tests and logistical challenges [14].

Earlier this year, the Philippines launched a pilot project to vaccinate nine-year-old children in public schools in three regions of the country [19]. Brazil just announced a dengue vaccination program in the southern state of Paraná [20]. This slow rollout of CYD-TDV is far from the enthusiastic uptake expected when a dengue vaccine would finally become available [21]. There is no question that a dengue vaccine is urgently needed, but countries are justifiably cautious of CYD-TDV.

The current debate surrounding CYD-TDV use highlights the urgent need for a better dengue vaccine. Of the dengue vaccine candidates, two are furthest along in clinical development [22]. The Takeda tetravalent dengue vaccine has completed a Phase II clinical trial in Puerto Rico, Colombia, Singapore, and Thailand [23] and is currently undergoing a multicountry Phase III evaluation [24]. The attenuated tetravalent lyophilized dengue vaccine developed by the United States National Institutes of Health and National Institute of Allergy and Infectious Diseases and manufactured by Butantan Institute has completed Phase I and II studies [25,26], and a Phase III [27] study is currently underway. Several other dengue vaccine candidates are in preclinical development. There remains hope that a dengue vaccine that confers simultaneous protection against all dengue serotypes will become available one day.

## Abbreviations

IgG: immunoglobulin G
PRNT: plaque reduction neutralization test
SAGE: Strategic Advisory Group of Experts
